# Editorial: Why was my manuscript rejected?

**DOI:** 10.1120/jacmp.v12i1.3558

**Published:** 2011-02-16

Over the past few years, the JACMP has received on the average over 200 proffered manuscripts per year. During this period, over 40% of these manuscripts were recommended for rejection by one of the Associate Editors. Authors receiving rejection letters are often disappointed, because they believe their work is worthy of publication – otherwise, they would not have taken the time and effort to write and submit the manuscript. Recently I reviewed a series of rejected manuscripts to determine the principal causes for rejection of a manuscript, identifying the pitfalls facing authors. By identifying the causes for rejection, I hope to encourage authors to avoid these pitfalls, and submit manuscripts that have a greater probability of acceptance.

The following are the four most common reasons why a JACMP submission was recommended for rejection by an Associate Editor:

1. The article was not sufficiently clinical.
  The JACMP is, first and foremost, a clinical journal. Although all in the medical physics community appreciate good science, if no clear clinical application of the science is presented in a manuscript, the manuscript is not appropriate for the JACMP. A work that has no clear clinical applications may be more appropriate for a journal with a more scientific orientation, such as *Medical Physics* or *Physics in Medicine and Biology*. We have seen some manuscript submissions in which the format presentation is very reminiscent of that required for other journals, leading us to the conclusion that the manuscript may have been submitted to a different journal, and then rejected. If the authors fail to recognize that the orientation of the JACMP is somewhat different from that of other journals and make no appropriate modifications in the manuscript, even if they resolve the issues that led to the manuscript's prior rejection, their manuscript is likely to be rejected by the JACMP as well. On the other hand, if the main reason for rejection by another journal was that the manuscript was highly clinical and did not introduce new science, then the JACMP may very well be the appropriate venue for this manuscript.
2. Nothing new was added by the study described in the article.
  The purpose of the JACMP is to introduce new clinical ideas and techniques into the medical physics community. If nothing new is added by the study, if the study is a repetition of work already done, if the results are obvious, then the referees are not likely to recommend acceptance of the manuscript. Furthermore, if the study is so specific that no one can use the information presented in the manuscript, then the JACMP will not be so presumptuous as to take up the valuable time of our readership with information they will not be able to use.
3. The article was not sufficiently rigorous.
  Even though the JACMP is a clinical journal, it is also a scientific journal, in that the basic principles of science still hold when applied to methodology. If a hypothesis is presented, it must be adequately tested, and include sufficient detail so that each step of the reasoning may be validated, and the reasoning process must be free from basic flaws. The methodology must be such that the results clearly follow. The results must be thoroughly analyzed, leading to well‐justified conclusions. Conjecture is acceptable, but only in the Discussion part of the manuscript, and only when it is clearly identified as such.
4. The English was poor.
  The JACMP is an international journal, and we encourage our overseas colleagues to share their knowledge with the North American medical physics community. Yet in some cases, the quality of the English language used in a manuscript is so poor that it prevents the reader from understanding the work of the authors. Unfortunately, the JACMP does not have the resources to rewrite a manuscript in high‐quality scientific English, so the onus for good language must be placed on the authors. The inability to write in good English is not limited to those for whom English is not their native tongue; on occasion, a request for rewriting has gone back to a native English‐speaking author. Poor English alone is not likely to cause rejection of a manuscript; language issues are often seen in combination with other problems. Fortunately, language issues are often the easiest problems to correct. However, even if the language is correct, the manuscript must still be made intelligible. (Please see my editorial from May 2009 for more details on how to write a good manuscript.)

We publish journals such as the JACMP as a means of communicating information to our colleagues. If our communication is poor – or worse, if there is no information to communicate – then we do our colleagues a disservice by pretending we have information to share. Fortunately, most of the manuscripts submitted to the JACMP are of high quality, are well‐written and, with the help of our Reviewers and Associate Editors, make it to final publication.

George Starkschall, PhD

Editor‐in‐Chief

February 15, 2011

